# Validation of the Japanese version of the Kenny Music Performance Anxiety Inventory-Revised

**DOI:** 10.3389/fpsyg.2025.1543958

**Published:** 2025-06-18

**Authors:** Sakie Takagi, Michiko Yoshie, Akihiko Murai

**Affiliations:** ^1^Department of Human and Engineered Environmental Studies, Graduate School of Frontier Sciences, The University of Tokyo, Kashiwa, Japan; ^2^Research Institute on Human and Societal Augmentation, National Institute of Advanced Industrial Science and Technology (AIST), Kashiwa, Japan; ^3^Human Informatics and Interaction Research Institute, National Institute of Advanced Industrial Science and Technology (AIST), Tsukuba, Japan

**Keywords:** music performance anxiety, K-MPAI, factor analysis, validation, anxiety inventory, musician

## Abstract

The Japanese version of the Kenny Music Performance Anxiety Inventory-Revised (K-MPAI-R) has been developed but not yet been validated. This study aims to validate and certify the Japanese version of the K-MPAI-R. Data were collected from 400 participants (250 men, 149 women, and one identifying as other), aged between 18 and 64 years (M = 46.84, SD = 10.45). The sample included 200 professional and 200 amateur musicians, comprising 309 instrumentalists and 91 vocalists. An exploratory factor analysis with promax rotation extracted seven factors that explained 55.8% of the total variance, demonstrating a structure similar to the original version. The scale showed high internal consistency, with a Cronbach's alpha of 0.93. Criterion-related validity was supported by correlations with the State-Trait Anxiety Inventory (r = 0.67) and Performance Anxiety Questionnaire (r = 0.75). These findings indicate that the Japanese version of the K-MPAI-R is a reliable and valid measure of music performance anxiety. This validated instrument enables further investigations into music performance anxiety among Japanese musicians.

## 1 Introduction

Music performance anxiety (MPA) is the experience of marked and persistent anxious apprehension related to musical performance, typically arising from specific anxiety conditioning experiences (Kenny, 2009b). MPA is accompanied by various symptoms, classified into the following three categories: physiological (e.g., increased heart rate, dry mouth, and sweating), mental (e.g., difficulty concentrating and memory-related issues), and behavioral (e.g., tremors and technical difficulties) (Burin and Osório, 2017; Steptoe, 2001; Salmon, 1990; Irie et al., 2023). MPA is a common issue among musicians (Fernholz et al., 2019) regardless of their cultural or national background. For example, 24% of musicians in Brazil reported experiencing MPA (Barbar et al., 2014). Van Kemenade et al. (1995) have found that 59% of musicians in a Dutch orchestra reported experiencing MPA, and Studer et al. (2011) have revealed that 22% of music students in Switzerland failed an exam because of MPA. Yoshie et al. (2011) have also identified MPA indicators in 64% of both professional and amateur musicians in Japan.

Various questionnaires have been developed to quantify an individual's level of MPA as a stable trait, often including items about physiological and psychological changes experienced in past performance situations (Yoshie and Morijiri, 2024). These questionnaires include the Kenny Music Performance Anxiety Inventory (K-MPAI and K-MPAI-R) (Kenny et al., 2004; Kenny, 2009a), Performance Anxiety Questionnaire (PAQ) (Cox and Kenardy, 1993), Mazzarolo Music Performance Anxiety Scale (Mazzarolo and Schubert, 2022) and Music Performance Anxiety Inventory for Adolescents (Osborne and Kenny, 2005). Some of such questionnaires, including the PAQ (Kobori et al., 2011) and the Competitive State Anxiety Inventory-2 for Musicians (Yoshie and Shigemasu, 2006; Yoshie et al., 2009), have been used to measure MPA levels among Japanese musicians. Although each questionnaire offers distinct strengths, the K-MPAI and K-MPAI-R (Kenny et al., 2004; Kenny, 2009a) have been widely adopted in research involving both professional and amateur musicians across various genres, instrumentalists, singers, and ensemble or orchestra participants (Robson and Kenny, 2017; Kenny et al., 2013; Kenny and Ackermann, 2015; Paliaukiene et al., 2018); it has also been translated into 22 languages (Kenny, 2023).

The K-MPAI was developed by Kenny et al. (2004), and is based on Barlow's emotion-based theory of anxiety (Barlow, 2000). Barlow (2000) has described the following three vulnerabilities related to the development of anxiety, anxiety disorders, and emotional disorders: generalized biological vulnerability, generalized psychological vulnerability, and specific psychological vulnerability. Generalized biological vulnerability describes a basic anxiety tendency driven by genetic influences. Generalized psychological vulnerability is shaped by early experiences with uncontrollability, which later amplify stressful events. Specific psychological vulnerability, influenced by early learning experiences, predisposes individuals to focus their anxiety on specific objects or events and influences which object or situation becomes the focus of fear in specific phobias. The K-MPAI comprises 26 items designed to assess such vulnerabilities indicated in Barlow's theory and pre-performance experience, aiming to contribute to the comprehensive conceptualization of MPA and provide an appropriate focus for the development of more suitable treatments (Kenny, 2009a).

Kenny later revised the K-MPAI, incorporating additional factors related to the etiology and maintenance of MPA with a broad focus. This led to the development of the K-MPAI-R with 40 items (Kenny, 2009a). Kenny et al. (2012) explored the factor structure of the K-MPAI-R using a sample of 377 professional orchestral musicians in Australia. A factor analysis identified the following six distinct factors: proximal somatic anxiety and worry about performance; worry/dread (negative cognitions/ruminations) focused on self/other scrutiny; depression/hopelessness (psychological vulnerability); parental empathy; concerns with memory; generational transmission of anxiety; and anxious apprehension and biological vulnerability, a weaker additional factor.

The K-MPAI-R has been translated into Spanish (Peru) (Chang-Arana et al., 2018), French (Antonini Philippe et al., 2022), Korean (Oh et al., 2020), Portuguese (Dias et al., 2022), Italian (Antonini Philippe et al., 2023), Polish (Kantor-Martynuska and Kenny, 2018), Turkish (Çiçek and Güdek, 2020) and Romanian (Faur et al., 2021), with reliability testing conducted through internal consistency coefficients. In addition, validity testing has been conducted through factor structure examination via exploratory factor analysis (EFA) (Chang-Arana et al., 2018; Antonini Philippe et al., 2022, 2023; Oh et al., 2020; Dias et al., 2022; Kantor-Martynuska and Kenny, 2018; Faur et al., 2021), and correlation analyses with related measures such as the State-Trait Anxiety Inventory (STAI) (Chang-Arana et al., 2018; Antonini Philippe et al., 2022, 2023; Oh et al., 2020; Dias et al., 2022; Kantor-Martynuska and Kenny, 2018). Among the factors derived by Kenny et al. (2012), “proximal somatic anxiety and worry about performance”, “depression/hopelessness (psychological vulnerability)”, “parental empathy”, and “concerns with memory” were also observed in a similar form across multiple language versions (Antonini Philippe et al., 2022, 2023; Dias et al., 2022; Faur et al., 2021; Chang-Arana et al., 2018; Oh et al., 2020; Kantor-Martynuska and Kenny, 2018). However, variations in the factor structure have also been found among different language versions of the K-MPAI-R. For example, factors related to “worry/dread (negative cognitions) focused on self/other scrutiny” were only found in French (Antonini Philippe et al., 2022), and Korean (Oh et al., 2020) versions. Factors related to “generational transmission of anxiety” were found only in Italian (Antonini Philippe et al., 2023) and Korean (Oh et al., 2020) versions. These results potentially indicate that differences in languages and/or cultures can influence the factor structure of the K-MPAI-R.

The various language versions of the K-MPAI-R have contributed to a better understanding of MPA, especially personality traits related to MPA. For example, a study conducted on Brazilian musicians found that the group with higher K-MPAI scores had lower self-assessment (Barbar et al., 2014). The K-MPAI-R was also used to evaluate the effectiveness of Acceptance and Commitment Therapy treatment on MPA management (Juncos et al., 2017).

The development of a Japanese version of the K-MPAI-R would lead to a deeper understanding of the characteristics of MPA among Japanese musicians and allow for comparisons with studies using other language versions. The authors have created the Japanese version of the K-MPAI-R (Kenny, 2023); however, it has yet to be validated. This study aims to develop a validated Japanese version of the K-MPAI-R. Responses from 400 musicians to the Japanese version of the K-MPAI-R were analyzed through the examination of both reliability (e.g., internal consistency) and validity (e.g., EFA). The validity of the Japanese version was assessed by comparing its factor structure with the English version (Kenny et al., 2012) and results from other language versions. Furthermore, its relationships with the STAI (Spielberger et al., 1970) and the PAQ (Cox and Kenardy, 1993) were examined.

## 2 Method

### 2.1 Measures

#### 2.1.1 Kenny Music Performance Anxiety Inventory Revised version (K-MPAI-R)

The K-MPAI was developed to assess anxiety symptoms and other associated constructs within the context of music performance. The original version includes 26 items (Kenny et al., 2004), which was later revised and expanded to include 40 items (Kenny Music Performance Anxiety Inventory Revised version: K-MPAI-R) (Kenny, 2009a). The questionnaire is answered on a seven-point Likert-type scale, ranging from 0 (“strongly disagree”) to 6 (“strongly agree”).

The Japanese version of the K-MPAI-R, developed by the authors through a back-translation process, was approved by Kenny (2023); however, it has yet to be validated. A revision of the Japanese version of the K-MPAI-R was conducted to identify any issues overlooked during the translation process and to improve the comprehensibility and cognitive equivalence of the scale. Established guidelines for scale translation recommend that revision processes include cognitive debriefing with multiple individuals from the target population (Wild et al., 2005). We therefore recruited seven musicians from the target population of the K-MPAIR, namely five professionals (a singer, pianist, trombonist, percussionist, and cellist), a university-level music student (a violist), and an amateur musician (a saxophonist), comprising four men and three women, including one bilingual speaker of English and Japanese. Following these interviews, the revisions were made with a focus on consistency with the original version and naturalness in Japanese. During this process, discussions were held among the authors, including experts in music psychology, to determine the final wording. Out of the 40 items, 22 were modified. These items were back-translated again to ensure consistency with the original version. The revised questionnaire is available in the Supplementary Data 1.

#### 2.1.2 State-Trait Anxiety Inventory (STAI)

The STAI (Spielberger et al., 1970) is a 40-item self-report questionnaire comprising 20 items each for trait anxiety and state anxiety, with responses provided on a four-point Likert-type scale, ranging from 1 (“not at all”) to 4 (“very much so”). Participants completed the Japanese version of the state scale of the STAI (Hidemi and Kuniharu, 1981). To assess their mental state during musical performances, the following instruction was added: “Imagine the most important performance you have had within the past five years and indicate how much you felt each of the following statements during that time.”

#### 2.1.3 Performance Anxiety Questionnaire (PAQ)

The PAQ (Cox and Kenardy, 1993) comprises 20 statements, with 10 describing cognitive feelings and 10 describing somatizations during musical performances. It measures how frequently participants experience these cognitive and somatic responses across the following three performance settings: practice, group public performances, and solo public performances. Participants rate each statement on a five-point Likert-type scale, ranging from 1 (“Never”) to 5 (“Always”) for each setting. All PAQ items were translated into Japanese through a back-translation process by Kobori et al. (2011). In this study, participants completed the Japanese version of the PAQ, responding to the statements specifically in the context of public performances, without distinguishing between solo and group performances.

### 2.2 Participants

A total of 400 individuals participated in this study. Among the participants, 250 were men, 149 were women, and one individual identified as other. The participants were between 18 and 64 years old, and their mean age was 46.84 years (SD = 10.45). All participants were native speakers of Japanese. Eligibility criteria required that participants be currently engaged in musical performance activities, specifically playing a musical instrument (*n* = 309) or singing (*n* = 91), and have given a public performance within the past five years. Public performances included situations where the performance was subject to evaluation, such as in music exams, competitions, or auditions, as well as performances before general audiences; however, it excluded performances limited to family, close friends, daily practice, classes, or lessons. The sample was evenly divided between professional (*n* = 200) and amateur (*n* = 200) musicians. The criteria for being classified as a professional were either (a) earning income from music or (b) having studied music at a university or specialized music school. This category also included school teachers with a music teaching license for junior high or high school or those who taught music as a specialized subject in elementary school.

### 2.3 Procedure

An online survey was conducted. The participants were recruited through an online panel maintained by a marketing research firm. Before participating, they read an explanation of the study and provided their informed consent. Those who consented were asked to complete the K-MPAI-R, STAI, and PAQ.

The study was conducted with the approval of the Ethics Committee of the National Institute of Advanced Industrial Science and Technology.

### 2.4 Data analysis

All participants answered all questions, and there was no missing data. Some items (1, 2, 9, 17, 23, 33, 35, 37) were reversed following Kenny (2009a). For the 40 items, means, standard deviations, skewness, and kurtosis were calculated. To assess the adequacy for factor analysis, the Kaiser-Meyer-Olkin (KMO) measure of sampling adequacy was calculated, and Bartlett's test of sphericity was conducted. An EFA with a maximum likelihood and promax rotation was conducted to examine the factor structure of the data. The results of the parallel analysis and Minimum Average Partial (MAP) were used as a reference for determining the number of factors. To assess the model fit, the Tucker-Lewis index (TLI) and root mean square error of approximation (RMSEA) were calculated. To assess scale reliability, the internal consistency coefficient, specifically Cronbach's alpha, was used. The procedures were developed by drawing on the methods of Antonini Philippe et al. (2022, 2023).

We calculated means and standard deviations for the STAI and PAQ. The items 1, 2, 5, 8, 10, 11, 15, 16, 19, and 20 were reversed for the STAI-state following Spielberger et al. (1970). The items 4 and 8 were reversed for the PAQ. To evaluate the reliability of the scales, we performed correlation analyses to investigate several key relationships. We calculated the correlations for the following using Pearson's correlation coefficient: the total scores of the K-MPAI-R and STAI; each factor score of the K-MPAI-R with the total score of the STAI; the total scores of the K-MPAI-R and PAQ; and each factor score of the K-MPAI-R with the total score of the PAQ.

Data analysis was conducted using R Core Team (2024) and psych package (v4.4.1; William Revelle, 2024).

## 3 Results

### 3.1 Descriptive analysis

The mean values and standard deviations for each item of the K-MPAI-R are shown in Table 1. Skewness and kurtosis coefficients were calculated for all 40 items. According to Tabachnick and Fidell (2007), skewness and kurtosis coefficients should be within ±1.5 when performing factor analysis on items measured using a Likert scale. The analysis indicates that all 40 items met this criterion.

**Table 1:** Descriptive statistics.

**Item**		**Mean**	**SD**	**Skewness**	**Kurtosis**
1*	I generally feel in control of my life.	2.65	1.58	0.43	−0.12
2*	I find it easy to trust others.	3.14	1.45	0.11	−0.23
3	Sometimes I feel depressed without knowing why.	2.72	1.80	0.08	−0.93
4	I often find it difficult to work up the energy to do things.	2.96	1.58	−0.15	−0.64
5	Excessive worrying is a characteristic of my family.	2.73	1.64	0.09	−0.65
6	I often feel that life has not much to offer me.	2.08	1.66	0.45	−0.64
7	Even if I work hard in preparation for a performance, I am likely to make mistakes.	3.17	1.57	−0.15	−0.47
8	I find it difficult to depend on others.	3.32	1.63	−0.04	−0.73
9*	My parents were mostly responsive to my needs.	2.74	1.65	0.26	−0.59
10	Prior to, or during a performance, I get feelings akin to panic.	2.08	1.63	0.31	−0.89
11	I never know before a concert whether I will perform well.	3.31	1.61	−0.22	−0.48
12	Prior to, or during a performance, I experience dry mouth.	2.84	1.67	−0.07	−0.73
13	I often feel that I am not worth much as a person.	2.42	1.76	0.31	−0.78
14	During a performance I find myself thinking about whether I'll even get through it.	2.56	1.78	0.09	−0.98
15	Thinking about the evaluation I may get interferes with my performance.	2.36	1.66	0.18	−0.83
16	Prior to, or during a performance, I feel sick or faint or have a churning in my stomach.	1.91	1.67	0.47	−0.79
17*	Even in the most stressful performance situations, I am confident that I will perform well.	3.42	1.60	−0.20	−0.60
18	I am often concerned about a negative reaction from the audience.	2.80	1.68	0.09	−0.72
19	Sometimes I feel anxious for no particular reason.	2.60	1.74	0.11	−0.88
20	From early in my music studies, I remember being anxious about performing.	2.43	1.65	0.19	−0.75
21	I worry that one bad performance may ruin my career.	2.27	1.83	0.35	−0.87
22	Prior to, or during a performance, I experience increased heart rate like pounding in my chest.	3.10	1.67	0.00	−0.72
23*	My parents almost always listened to me.	2.79	1.63	0.10	−0.64
24	I give up worthwhile performance opportunities.	2.34	1.66	0.21	−0.80
25	After the performance, I worry about whether I played well enough.	3.14	1.66	−0.15	−0.69
26	My worry and nervousness about my performance interferes with my focus and concentration.	2.56	1.58	0.06	−0.72
27	As a child, I often felt sad.	2.77	1.71	0.08	−0.78
28	I often prepare for a concert with a sense of dread and impending disaster.	2.39	1.74	0.22	−0.87
29	One or both of my parents were overly anxious.	2.28	1.69	0.31	−0.75
30	Prior to, or during a performance, I have increased muscle tension.	2.66	1.64	0.08	−0.63
31	I often feel that I have nothing to look forward to.	2.29	1.73	0.27	−0.82
32	After the performance, I replay it in my mind over and over.	2.91	1.65	0.10	−0.63
33*	My parents encouraged me to try new things.	3.06	1.61	0.15	−0.59
34	I worry so much before a performance, I cannot sleep.	2.32	1.65	0.18	−0.85
35*	When performing without music, my memory is reliable.	2.73	1.61	0.10	−0.68
36	Prior to, or during a performance, I experience shaking or trembling or tremor.	2.47	1.69	0.22	−0.72
37*	I am confident playing from memory.	3.21	1.66	−0.11	−0.59
38	I am concerned about being scrutinized by others.	2.93	1.69	−0.04	−0.69
39	I am concerned about my own judgment of how I will perform.	3.08	1.62	−0.07	−0.48
40	I remain committed to performing even though it causes me great anxiety.	3.50	1.59	−0.03	−0.57

*Reversed scored items.

### 3.2 Exploratory factor analysis

The KMO assesses sampling adequacy. The KMO value was 0.93, indicating excellent adequacy. Furthermore, the KMO values for each item were above 0.6, confirming the suitability of the data for factor analysis. Bartlett's test of sphericity also confirmed the data's adequacy, with χ^2^(780) = 9, 593.6, *p < * 0.001.

A factor analysis using maximum likelihood estimation and promax rotation was performed on the 40 items of the K-MPAI-R. To determine the number of factors, both parallel analysis and the MAP criterion were used. The parallel analysis suggested a seven-factor solution, while the MAP criterion recommended five factors. Accordingly, EFA was conducted for the five-, six-, and seven-factor models. The fit indices for the models were as follows: for the five-factor solution, χ^2^(590) = 1, 508.05, *p < * 0.001, ratio χ^2^/*df =* 2.56, TLI = 0.86 and RMSEA = 0.062; for the six-factor solution, χ^2^(555) = 1, 283.15, *p < * 0.001, ratio χ^2^/*df =* 2.31, TLI = 0.88 and RMSEA = 0.057; and for the seven-factor solution, χ^2^(521) = 1, 127.42, *p < * 0.001, ratio χ^2^/*df =* 2.16, TLI = 0.90 and RMSEA = 0.054. A TLI value above 0.90 and RMSEA value below 0.08 are generally considered acceptable (Bader and Moshagen, 2022). Considering both the fit indices and content of each factor, the seven-factor solution was determined to be the most appropriate.

The factors were named based on the items with factor loadings of 0.40 or higher.

F1: Music performance anxiety symptoms (10 items: 10, 12, 15, 16, 22, 24, 26, 30, 34, 36; α* =* 0.91);
F2: Psychological vulnerability (8 items: 3, 4, 6, 8, 13, 19, 20, 31; α* =* 0.89);
F3: Worry/dread focused on self/other scrutiny and evaluation (6 items: 18, 21, 25, 28, 38, 39; α* =* 0.89);
F4: Parental support (3 items: 9, 23, 33; α* =* 0.79);
F5: Memory and self-efficacy (3 items: 17, 35, 37; α* =* 0.67).
F6: Uncontrollability (2 items: 7, 11; α* =* 0.75).
F7: Generational transmission of anxiety (2 items: 5, 29; α* =* 0.67).

We calculated Cronbach's alpha to measure internal consistency. The alphas for factors 1 through 7 were 0.91, 0.89, 0.89, 0.79, 0.67, 0.75, and 0.67, respectively, indicating good reliability for each factor. The overall scale had a reliability of α = 0.93.

The seven factors explained 55.8% of variance (Table 2). The correlations between the seven factors are provided in the Supplementary Figure 1.

**Table 2:** Factor loadings of the K-MPAI-R Japanese version.

**Item**		**Factor 1 Music performance anxiety symptom**	**Factor 2 Psychological vulnerability**	**Factor 3 Worry/dread on self/other scrutiny and evaluation**	**Factor 4 Parental support**	**Factor 5 Memory and self-efficacy**	**Factor 6 Uncontrollability**	**Factor 7 Generational transmission of anxiety**	**Communality**
36	Prior to, or during a performance, I experience shaking or trembling or tremor.	**0.769**	−0.130	0.081	0.037	−0.049	0.066	−0.062	0.551
30	Prior to, or during a performance, I have increased muscle tension.	**0.748**	−0.187	0.110	0.080	−0.026	0.154	0.041	0.585
10	Prior to, or during a performance, I get feelings akin to panic.	**0.737**	0.095	−0.198	−0.025	0.167	0.170	0.129	0.589
16	Prior to, or during a performance, I feel sick or faint or have a churning in my stomach.	**0.736**	0.352	−0.285	−0.020	0.000	−0.103	−0.038	0.708
12	Prior to, or during a performance, I experience dry mouth.	**0.608**	0.125	−0.155	−0.091	−0.005	0.267	−0.193	0.415
22	Prior to, or during a performance, I experience increased heart rate like pounding in my chest.	**0.599**	−0.208	0.197	0.005	−0.046	0.311	−0.057	0.569
34	I worry so much before a performance, I cannot sleep.	**0.598**	0.136	0.100	−0.025	−0.035	−0.105	0.017	0.587
26	My worry and nervousness about my performance interferes with my focus and concentration.	**0.504**	−0.011	0.360	0.045	0.055	0.055	0.135	0.686
15	Thinking about the evaluation I may get interferes with my performance.	**0.452**	0.276	0.183	−0.087	0.169	0.045	0.031	0.678
24	I give up worthwhile performance opportunities.	**0.430**	0.331	−0.010	0.097	−0.154	−0.102	−0.016	0.488
4	I often find it difficult to work up the energy to do things.	−0.118	**0.825**	−0.058	−0.032	−0.100	0.269	−0.022	0.585
13	I often feel that I am not worth much as a person.	0.089	**0.770**	−0.049	0.078	0.019	0.137	−0.014	0.674
3	Sometimes I feel depressed without knowing why.	−0.169	**0.765**	0.053	0.020	−0.049	0.223	0.015	0.539
6	I often feel that life has not much to offer me.	0.111	**0.739**	−0.223	0.057	0.036	0.005	0.132	0.595
19	Sometimes I feel anxious for no particular reason.	0.113	**0.661**	0.144	−0.024	−0.074	0.146	−0.140	0.684
31	I often feel that I have nothing to look forward to.	0.120	**0.639**	−0.023	−0.022	−0.068	−0.046	0.146	0.603
20	From early in my music studies, I remember being anxious about performing.	0.322	**0.499**	0.018	−0.066	−0.070	−0.075	−0.022	0.579
8	I find it difficult to depend on others.	−0.079	**0.403**	0.067	0.086	−0.067	0.391	−0.015	0.330
38	I am concerned about being scrutinized by others.	−0.043	0.023	**0.834**	−0.069	−0.041	−0.053	0.063	0.667
39	I am concerned about my own judgment of how I will perform.	0.053	−0.128	**0.790**	−0.039	0.007	0.149	0.040	0.703
25	After the performance, I worry about whether I played well enough.	0.038	−0.044	**0.725**	0.021	0.047	0.104	0.126	0.623
28	I often prepare for a concert with a sense of dread and impending disaster.	0.390	0.069	**0.469**	0.052	−0.026	−0.144	0.093	0.681
18	I am often concerned about a negative reaction from the audience.	0.129	0.335	**0.434**	−0.197	−0.004	0.016	−0.164	0.592
21	I worry that one bad performance may ruin my career.	0.301	0.179	**0.417**	−0.021	−0.040	−0.152	0.119	0.637
9*	My parents were mostly responsive to my needs.	0.176	−0.116	−0.117	**0.835**	−0.020	−0.028	0.025	0.659
23*	My parents almost always listened to me.	−0.008	0.085	−0.218	**0.805**	0.008	0.051	0.063	0.655
33*	My parents encouraged me to try new things.	−0.218	0.096	0.024	**0.565**	0.145	0.013	−0.060	0.493
35*	When performing without music, my memory is reliable.	−0.003	−0.024	−0.044	−0.012	**0.712**	−0.080	0.151	0.481
37*	I am confident playing from memory.	0.002	−0.073	−0.036	0.057	**0.674**	0.184	0.023	0.457
17*	Even in the most stressful performance situations, I am confident that I will perform well.	−0.030	−0.165	0.245	0.086	**0.548**	0.199	−0.153	0.476
11	I never know before a concert whether I will perform well.	0.388	0.110	0.126	0.042	0.117	**0.545**	0.003	0.695
7	Even if I work hard in preparation for a performance, I am likely to make mistakes.	0.048	0.306	0.110	−0.097	0.319	**0.497**	0.143	0.553
29	One or both of my parents were overly anxious.	0.171	0.101	0.220	0.041	0.051	−0.106	**0.596**	0.622
5	Excessive worrying is a characteristic of my family.	−0.120	0.357	0.132	−0.089	0.187	0.129	**0.505**	0.486
1*	I generally feel in control of my life.	0.032	0.113	0.068	0.127	0.142	−0.208	−0.294	0.242
14	During a performance I find myself thinking about whether I'll even get through it.	0.380	0.327	0.202	−0.083	0.156	0.060	0.006	0.656
2*	I find it easy to trust others.	−0.006	−0.020	0.173	0.377	0.074	−0.005	−0.164	0.254
27	As a child, I often felt sad.	0.118	0.192	0.181	0.300	−0.206	0.166	0.179	0.411
32	After the performance, I replay it in my mind over and over.	0.186	0.131	0.193	−0.030	−0.205	0.186	0.100	0.397
40	I remain committed to performing even though it causes me great anxiety.	−0.098	−0.113	0.274	−0.088	−0.437	0.239	−0.030	0.418
	Percentage of variance explained	15.1	13.8	9.9	5.2	4.5	4.2	3.0	

Factor loadings greater than 0.4 are in bold. *Reversed scored items.

The six items with factor loadings of less than 0.4 (1, 2, 14, 27, 32, and 40) were not included in any factor. Similarly to previous literature (Antonini Philippe et al., 2023), we used the total score of all 40 items in the subsequent analyses, rather than refining the scale by removing items.

### 3.3 K-MPAI-R and STAI

The average score of the STAI-state was 46.67 (SD = 10.35) and Cronbach's alpha was 0.89. The K-MPAI-R scores were positively correlated with the STAI score (Table 3).

**Table 3:** Descriptive statistics and correlations between K-MPAI-R factors (seven factors and total score) and STAI.

**Variable**	**Number of items**	**M (SD)**	**Correlation with STAI**
F1	10	24.64 (12.23)	0.58***
F2	8	20.83 (10.17)	0.52***
F3	6	16.60 (8.21)	0.59***
F4	3	8.60 (4.11)	0.04***
F5	3	12.86 (3.5)	0.26***
F6	2	6.48 (2.84)	0.48***
F7	2	5.01 (2.89)	0.40***
KMPAI-R	40	109.04 (34.63)	0.67***
STAI	20	46.67 (10.35)	–

F1:Music performance anxiety symptoms, F2:Psychological vulnerability, F3:Worry/dread on self/other scrutiny and evaluation, F4:Parental support, F5:Memory and self-efficacy, F6:Uncontrollability, F7:Generational transmission of anxiety.

*** *p < * 0.001.

### 3.4 K-MPAI-R and PAQ

The average score of PAQ was 54.24 (SD = 14.53) and Cronbach's alpha was 0.94. The K-MPAI-R scores were positively correlated with the PAQ score (Table 4).

**Table 4:** Descriptive statistics and correlations between K-MPAI-R factors (seven factors and total score) and PAQ.

**Variable**	**Number of items**	**M (SD)**	**Correlation with PAQ**
F1	10	24.64 (12.23)	0.75***
F2	8	20.83 (10.17)	0.58***
F3	6	16.60 (8.21)	0.64***
F4	3	8.60 (4.11)	− 0.03***
F5	3	12.86 (3.50)	0.11*
F6	2	6.48 (2.84)	0.52***
F7	2	5.01 (2.89)	0.41***
KMPAI-R	40	109.04 (34.63)	0.75***
PAQ	20	54.24 (14.53)	–

F1:Music performance anxiety symptoms, F2:Psychological vulnerability, F3:Worry/dread on self/other scrutiny and evaluation, F4:Parental support, F5:Memory and self-efficacy, F6:Uncontrollability, F7:Generational transmission of anxiety.

* *p < * 0.05. *** *p < * 0.001.

## 4 Discussion

This study developed a validated Japanese version of the K-MPAI-R. The results demonstrated that the developed questionnaire is reliable for measuring MPA. This conclusion is supported by a Cronbach's alpha coefficient of 0.93, which indicates strong internal consistency.

The Japanese version of the K-MPAI-R showed a moderate level of correlation with the STAI-State (*r* = 0.67, *p* < 0.001), indicating its construct validity. The results are consistent with previous studies that showed moderate levels of correlations (r = 0.52 − 0.79) between other language versions of the K-MPAI-R and STAI-State (Antonini Philippe et al., 2022, 2023; Dias et al., 2022). The Japanese version of the K-MPAI-R also showed a moderate level of correlation with the PAQ (r = 0.75, *p* < 0.001). Since the PAQ measures the frequency of cognitive and somatic responses experienced during musical performances, its correlation with the K-MPAI-R further reinforces its criterion-related validity. The correlation was particularly strong for factors directly related to public performance, such as F1 and F3. These findings highlight the positive relationships between the K-MPAI-R and other measures of anxiety, strengthening the instrument's validity.

The factor structure of the Japanese version of the K-MPAI-R was derived through EFA. Factor 1, “Music Performance Anxiety Symptoms,” includes both somatic symptoms (Items 36, 30, 16, 12, and 22) and cognitive symptoms (Items 15, 26, 34, and 10) that appear before or during a performance. Factor 2, labeled “Psychological Vulnerability,” includes items related to low self-esteem (Items 6 and 13), lack of energy or motivation (Items 4 and 31), vague or unexplained anxiety (Items 3 and 19), performance-related anxiety (Item 20), and difficulty depending on others (Item 8). Factor 3, titled “Worry/Dread Focused on Self/Other Scrutiny and Evaluation,” contains items reflecting traits related to a general concern about being evaluated by others (Items 18, 38, and 39) and behaviors or emotions driven by the fear and worry associated with scrutiny (Items 21, 25, and 28). Factor 4, “Parental Support,” concerns whether parents were supportive and responsive, specifically regarding their responsiveness to needs, active listening, and encouragement for trying new things. Factor 5, “Memory and Self-Efficacy,” reflects a sense of confidence in one's memory and ability to perform well, even in stressful environments. Factor 6, “Uncontrollability,” expresses uncertainty about performance outcomes and the likelihood of making mistakes, regardless of effort or preparation. Factor 7, “Generational Transmission of Anxiety,” comprises items 5 and 29.

The factor structure of the Japanese version of the K-MPAI-R was generally consistent with other language versions, including English (Kenny, 2009a; Kenny et al., 2012), Spanish (Peru) (Chang-Arana et al., 2018), French (Antonini Philippe et al., 2022), Korean (Oh et al., 2020), Portuguese (Dias et al., 2022), Italian (Antonini Philippe et al., 2023), Polish (Kantor-Martynuska and Kenny, 2018) and Romanian (Faur et al., 2021). Among the extracted factors, Factors 1, 2, 4, and 5 were globally shared across multiple language versions. In addition, for the remaining factors, each had corresponding factors in other language versions (Supplementary Table 1). There were no factors derived only in the Japanese version. These findings suggest a consistency in the factor structure of the K-MPAI-R across languages. Overall, the factor structure of the Japanese version of the K-MPAI-R is closely aligned with that identified by Kenny et al. (2012). A comparison of the factors and the items they include can be found in the Supplementary Table 2.

This study involved 400 participants, including 309 musical instrument players and 91 singers, with an equal distribution between professional and amateur musicians. The results obtained from this diverse sample showed that the internal consistency, factor structure, construct validity, and criterion-related validity of the K-MPAI-R were all sufficient, demonstrating the reliability of the Japanese version of the K-MPAI-R. However, this study has several limitations. First, the Japanese version of the K-MPAI-R was validated using a sample of adults aged 18–64 years. Therefore, further investigation is needed to determine its applicability for individuals under 18. Second, while the factor structure of the Japanese version was generally consistent with the original English and other language versions, several differences were observed in the identified factors and/or the items included in them (Supplementary Table 2). Similar discrepancies can also be found between the English and other language versions (Supplementary Table 1). Future research should explore the factors underlying these differences, including potential cultural influences. Third, the present study collected responses from a broad sample, including both amateur and professional musicians, as well as instrumentalists and singers. Further research should analyze the K-MPAI-R scores within specific subgroups to explore individual characteristics associated with vulnerability to MPA.

Understanding MPA and its related factors in Japanese musicians using the K-MPAI-R may provide insights into both globally shared and Japanese-specific mechanisms underlying MPA. The Japanese version of the K-MPAI-R would lead to a deeper understanding of the prevalence and characteristics of MPA among Japanese musicians, contributing to the development of more effective interventions and support systems tailored to their needs.

## Data Availability

The deidentified questionnaire data supporting the conclusions of this article will be made available by the corresponding authors upon request. Further inquiries can be directed to the corresponding authors.
