# Supplementary material for: Validation of the Japanese version of the Kenny Music Performance Anxiety Inventory-Revised
Source: Front Psychol. 2025 Jun 18;16:1543958. doi: 10.3389/fpsyg.2025.1543958 (PMC12213870; doi:10.3389/fpsyg.2025.1543958)
Supplement: Supplementary file 1 [file Table_1.docx]

Supplementary Material

Validation of the Japanese version of the Kenny Music Performance Anxiety Inventory-Revised

Sakie Takagi^*^, Michiko Yoshie^*^ and Akihiko Murai.

* Correspondence Sakie Takagi [sakie.takagi@aist.go.jp](mailto:sakie.takagi@aist.go.jp), Michiko Yoshie [m.yoshie@aist.go.jp](mailto:m.yoshie@aist.go.jp)

# Supplementary Data

**Supplementary Data 1:** *Japanese version of the Kenny Music Performance Anxiety Inventory-Revised (K-MPAI-R)* File name: Data Sheet 1.pdf

# Supplementary Figures and Tables

## Supplementary Figures


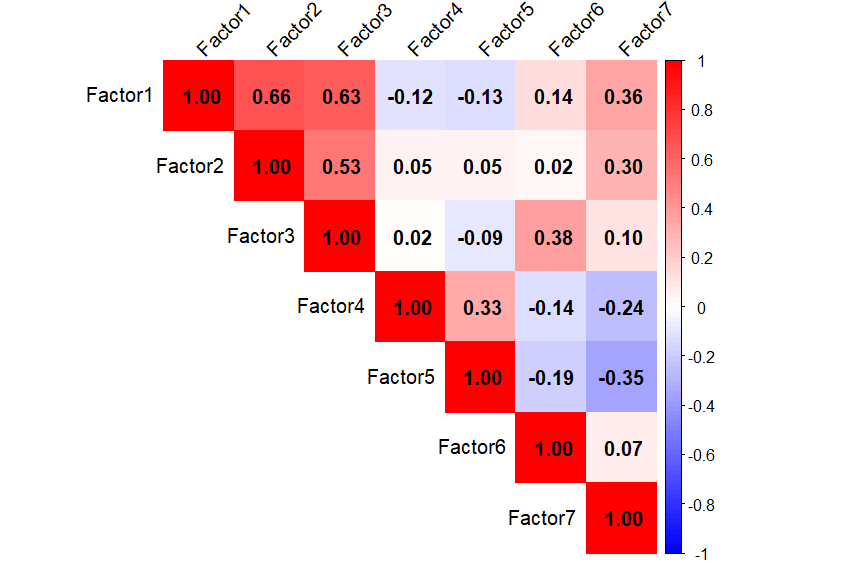


**Supplementary Figure 1.** The correlations between the seven factors of the K-MPAI-R Japanese version. Factor 1: Music performance anxiety symptoms, Factor2: Psychological vulnerability, Factor3: Worry/dread on self/other scrutiny and evaluation, Factor4: Parental support, Factor 5: Memory and self-efficacy, Factor 6: Uncontrollability, Factor 7: Generational transmission of anxiety.

## Supplementary Tables

| **The factor of Japanese version.** | **English (Kenny et al., 2012)** | **English (Kenny, 2009a)** | **Italian (Antonini Philippe et al., 2023)** | **French (Antonini Philippe et al., 2022)** | **Portuguese (Dias et al., 2022)** | **Romanian (Faur et al., 2021)** | **Spanish (Peru) (Chang-Arana et al., 2018)** | **Korean (Oh et al., 2020)** | **Polish (Kantor-Martynuska and Kenny, 2018)** |
| --- | --- | --- | --- | --- | --- | --- | --- | --- | --- |
| F1: Music performance anxiety symptoms | Yes | Yes | Yes | Yes | Yes | Yes | Yes | Yes | Yes |
| F2: Psychological vulnerability | Yes | Yes | Yes | Yes | Yes | Yes | Yes | Yes | Yes |
| F3: Worry/dread on self/other scrutiny and evaluation | Yes | Yes | No | Yes | No | No | No | Yes | No |
| F4: Parental support | Yes | Yes | Yes | Yes | Yes | Yes | No | Yes | Yes |
| F5: Memory and self-efficacy | Yes | Yes | Yes | Yes | Yes | Yes | No | No | No |
| F6: Uncontrollability | No | Yes | No | No | No | No | No | No | No |
| F7: Generational transmission of anxiety | Yes | Yes | Yes | No | No | No | No | No | No |

**Supplementary Table 1.** Correspondence of Factors Between the Japanese Version and Other Language Versions of the K-MPAI-R

Yes: The language version has a factor similar to the factor in the Japanese version. No: The language version doesn’t have a factor similar to the factor in the Japanese version.

**Supplementary Table 2.** A comparison of the factors and items in the Japanese version of the K-MPAI-R and the original version (Kenny, 2012).

| **Factor of Japanese ver.** | **items** | **Factor of English ver.** | **items** |
| --- | --- | --- | --- |
| Music performance anxiety symptoms | 10 | Proximal somatic anxiety and worry about performance | 10 |
|  | 12 |  | 12 |
|  | 16 |  | 16 |
|  | 22 |  | 22 |
|  | 26 |  | 26 |
|  | 30 |  | 30 |
|  | 34 |  | 34 |
|  | 36 |  | 36 |
|  | 15 |  | 14 |
|  | 24 |  | 28 |
|  |  |  | 40 |
| Psychological vulnerability | 3 | Depression/hopelessness (Psychological vulnerability) | 3 |
|  | 4 |  | 4 |
|  | 6 |  | 6 |
|  | 8 |  | 8 |
|  | 13 |  | 13 |
|  | 31 |  | 31 |
|  | 19 |  | 1 |
|  | 20 |  | 2 |
|  |  | Biological vulnerability | 20 |
| Worry/dread on self/other scrutiny and evaluation | 18 | Worry/dread (Negative cognitions) focused on self/other scrutiny | 18 |
|  | 21 |  | 21 |
|  | 25 |  | 25 |
|  | 38 |  | 38 |
|  | 39 |  | 39 |
|  | 7 |  | 28 |
|  | 15 |  |  |
|  | 32 |  |  |
| Parental support | 9 | Parental empathy | 9 |
|  | 23 |  | 23 |
|  | 33 |  | 33 |
|  |  |  | 27 |
| Memory and self-efficacy | 35 | Memory | 35 |
|  | 37 |  | 37 |
|  | 17 |  |  |
| Generational transmission of anxiety | 5 | Generational transmission of anxiety | 5 |
|  | 29 |  | 29 |
|  |  |  | 19 |
| Uncontrollability | 7 |  |  |
|  | 11 |  |  |
|  |  | Anxious apprehension | 11 |
|  |  |  | 17 |
|  |  |  | 24 |
| factor loadings < 0.4 | 1 |  |  |
|  | 2 |  |  |
|  | 14 |  |  |
|  | 27 |  |  |
|  | 32 |  |  |
|  | 40 |  |  |

Kenny, D., Driscoll, T., and Ackermann, B. (2012). Psychological well-being in professional orchestral musicians in australia: A descriptive population study. Psychol. Music 42, 210–232. doi:10.1177/0305735612463950.
