# Supplementary material for: Validation of the Japanese version of the Kenny Music Performance Anxiety Inventory-Revised
Source: Front Psychol. 2025 Jun 18;16:1543958. doi: 10.3389/fpsyg.2025.1543958 (PMC12213870; doi:10.3389/fpsyg.2025.1543958)
Supplement: Supplementary file 2 [file Data_Sheet_1.pdf]

Japanese version of the Kenny Music Performance Anxiety Inventory-Revised（K-MPAI-R）

以下は、一般的な心境と、演奏前・演奏中の心境についての文章です。それぞれの文章について１つの数字を選び、どの程度あなたにあてはまるかを答えてください。

		全く あてはまらない						非常に あてはまる
K_1	概して自分の人生をコントロールしていると感じる。	0	1	2	3	4	5	6
K_2	他人を信頼することは簡単だと思う。	0	1	2	3	4	5	6
K_3	訳も分からず落ち込むことが時々ある。	0	1	2	3	4	5	6
K_4	物事を行う気力が湧きづらいと思うことがよくある。	0	1	2	3	4	5	6
K_5	自分の家族は心配し過ぎる傾向がある。	0	1	2	3	4	5	6
K_6	人生から得るものはあまりないと感じることがよくある。	0	1	2	3	4	5	6
K_7	演奏に向けて懸命に準備しても、自分はミスをしがちである。	0	1	2	3	4	5	6
K_8	他人に頼ることが苦手だと思う。	0	1	2	3	4	5	6
K_9	自分の両親は、ほぼ自分の要求に応えてくれた。	0	1	2	3	4	5	6
K_10	演奏前や演奏中に、パニックに近い感覚になる。	0	1	2	3	4	5	6
K_11	コンサート前はいつも、自分がうまく演奏できるかどうか分からない。	0	1	2	3	4	5	6
K_12	演奏前や演奏中に、口の渇きを経験する。	0	1	2	3	4	5	6
K_13	自分はあまり価値のない人間だと感じることがよくある。	0	1	2	3	4	5	6
K_14	演奏中に、自分がやり通せるかどうかとすら考えてしまう。	0	1	2	3	4	5	6
K_15	自分が受けるかもしれない評価について考えることによって、自分の演奏が妨げられる。	0	1	2	3	4	5	6
K_16	演奏前や演奏中に、気分が悪くなったり、気が遠くなったり、胃がむかむかしたりする。	0	1	2	3	4	5	6
K_17	非常にストレスがかかる演奏場面ですら、自分はうまく演奏できる自信がある。	0	1	2	3	4	5	6
K_18	聴き手のネガティブな反応が気になることがよくある。	0	1	2	3	4	5	6
K_19	特に訳もなく不安を感じることが時々ある。	0	1	2	3	4	5	6
K_20	音楽を学び始めた頃から、演奏することに不安を感じていたのを覚えている。	0	1	2	3	4	5	6

		全く あてはまらない						非常に あてはまる
K_21	一度の演奏の失敗で自分のキャリアが台無しになるかもしれないと心配する。	0	1	2	3	4	5	6
K_22	演奏前や演奏中に、胸がドキドキするような心拍数の増加を経験する。	0	1	2	3	4	5	6
K_23	自分の両親は、ほぼいつも自分の話を聞いてくれた。	0	1	2	3	4	5	6
K_24	貴重な演奏の機会を諦めることがある。	0	1	2	3	4	5	6
K_25	演奏後に、自分が十分にうまく演奏できたかどうか心配になる。	0	1	2	3	4	5	6
K_26	自分の演奏に関する心配と緊張によって、集中が妨げられる。	0	1	2	3	4	5	6
K_27	子供の頃、悲しみを感じることがよくあった。	0	1	2	3	4	5	6
K_28	恐怖感と、最悪の事態が起こりそうな感覚を抱きながら、コンサートの準備をすることがよくある。	0	1	2	3	4	5	6
K_29	自分の片親または両親は、不安になり過ぎる傾向があった。	0	1	2	3	4	5	6
K_30	演奏前や演奏中に、筋肉の緊張が増す。	0	1	2	3	4	5	6
K_31	楽しみにしていることが何もないと感じることがよくある。	0	1	2	3	4	5	6
K_32	演奏後に、何度も繰り返し演奏を思い起こす。	0	1	2	3	4	5	6
K_33	自分の両親は、新しい物事に挑戦するよう、自分に勧めてくれた。	0	1	2	3	4	5	6
K_34	演奏前に、心配し過ぎて眠れない。	0	1	2	3	4	5	6
K_35	暗譜での演奏中に、自分の記憶は頼りになる。	0	1	2	3	4	5	6
K_36	演奏前や演奏中に、身体の震えを経験する。	0	1	2	3	4	5	6
K_37	暗譜で演奏することに自信がある。	0	1	2	3	4	5	6
K_38	他人に評価されていることが気になる。	0	1	2	3	4	5	6
K_39	自分がどの程度うまく演奏できると思えるかが、気になる。	0	1	2	3	4	5	6
K_40	強い不安を感じても、演奏することに今後も専念していく。	0	1	2	3	4	5	6

©Kenny, D.T. (2009). *Kenny Music Performance Anxiety Inventory-Revised* (K-MPAI-R)
Translated by Sakie Takagi, Michiko Yoshie, and Akihiko Murai
